# Surface plasmon resonance immunosensor for label-free detection of BIRC5 biomarker in spontaneously occurring canine mammary tumours

**DOI:** 10.1038/s41598-019-49998-x

**Published:** 2019-09-17

**Authors:** Subas Chandra Jena, Sameer Shrivastava, Sonal Saxena, Naveen Kumar, Swapan Kumar Maiti, Bishnu Prasad Mishra, Raj Kumar Singh

**Affiliations:** 1 Facility for Research and Training on Bioassays and Biosensor, Division of Veterinary Biotechnology, ICAR-Indian Veterinary Research Institute [Deemed University], Izatnagar 243122, India; 2 Division of Surgery, ICAR-Indian Veterinary Research Institute [Deemed University], Izatnagar, 243122 India

**Keywords:** Cancer screening, Diagnostic markers

## Abstract

We report detection of Baculoviral inhibitor of apoptosis repeat containing-5 (BIRC5) protein biomarker in dog serum by label-free surface plasmon resonance (SPR) immunosensor. Initially, overexpression of BIRC5 in canine mammary tumour (CMT) tissues was confirmed by real-time PCR. Recombinant BIRC5 was produced and protein specific antibodies developed in guinea pig specifically reacted with native protein in immunohistochemistry and immunocytochemistry. SPR immunosensor was developed by fabricating anti-BIRC5 antibodies on gold sensor disc. The equilibrium dissociation constant, (K_D_ = k_d_/k_a_) was 12.1 × 10^−12^ M; which indicates that antibodies are of high affinity with sensitivity in picomolar range. The SPR assay could detect as low as 6.25 pg/ml of BIRC5 protein in a calibration experiment (r^2^ = 0.9964). On testing real clinical samples, 95% specificity and 73.33% sensitivity were recorded. The average amount of serum BIRC5 in dogs with CMT was 110.02 ± 9.77 pg/ml; whereas, in non-cancerous disease conditions, 44.79 ± 4.28 pg/ml and in healthy dog sera 30.28 ± 2.99 pg/ml protein was detected. The SPR immunosensor for detection of BIRC5 in dog sera is reported for the first time and this may find prognostic and diagnostic applications in management of CMT. In future, ‘on-site’ sensors can be developed using this technique for near-patient testing.

## Introduction

Biosensor is a device that integrates biological recognition element like protein, nucleic acid, antibody or enzyme as capture agent which interact with analyte of interest and generate measurable signal through a transduction element. In the past few years, surface plasmon resonance (SPR) technique has been used extensively to monitor interaction between two binding molecules and detect pathogens including bacteria and viruses^1^, nucleic acids^2^, toxins^3–5^, allergens^6^, teliosporic antigen^7^ and antibodies^8,9^. The SPR biosensor allows direct, real-time, label-free and quantitative detection of interactions by measuring refractive index change at or near a thin metal film surface^10,11^ and offer unique opportunity for rapid and cost effective detection and identification of target biomolecules^12^. Antibodies are the most preferred biomolecules for capture of any analyte in a test sample^13^. SPR sensors have been applied to clinical and medical diagnosis^14,15^, with research focusing on developing assays to measure antigen-antibody interactions. SPR is coming up as one of the most rapidly developing technique to aid detection of biomarkers and focuses on measuring even low concentrations of target analyte in a test sample.

The mammary gland tumour in canines, also called as canine mammary tumour (CMT) are amongst the most common neoplasms especially of female dogs with higher incidence in bitches ageing more than five years^16^. Majority of CMTs have poor clinical outcomes with mortality rates about three times higher than human breast cancer^17^. Thus CMT presents an important neoplastic disease condition of dogs and is also a suitable model for human breast cancer biomarker discovery owing to similarity of CMT with human breast cancer. Early diagnosis is essential for effective treatment and monitoring of cancers^18,19^. In this direction, biomarkers associated with cancers are to be identified and simple, minimally non-invasive test for detecting biomarkers, preferably in body fluids needs to be developed^20^. But, low initial concentrations of these aberrant marker proteins in blood samples is a limiting factor for developing sensitive assays using conventional immunological techniques.

The baculoviral inhibitor of apoptosis repeat containing-5 (BIRC5), also called *survivin*, is the smallest member of inhibitor of apoptosis (IAP) family of proteins with anti-apoptotic, as well as, mitotic regulatory functions. BIRC5 inhibits apoptosis by interfering with the function of caspase-3, caspase-7, and caspase-9 and also act in caspase-independent pathways^21^. Suppression of apoptosis may initiate carcinogenesis in several ways. Although this protein is highly expressed in embryonic and foetal tissues, it is undetectable in terminally differentiated adult cells^22,23^. The overexpression of BIRC5 in human breast cancer and CMTs has been reported^24,25^ and autoantibodies to BIRC5 are also detected in human and dog sera^26^; but, a sensitive assay for detecting BIRC5 protein biomarker in dog serum is not yet reported.

During the past few years, BIRC5 has emerged as a biomarker of choice for early prediction of malignancies in humans^27^. BIRC5 overexpression has been reported in 34 of 67 (50.7%) breast cancer patients, but not in healthy women^28^. Besides breast cancer, BIRC5 detection has been related with prognosis and diagnosis of different human cancers such as acute lymphoblastic leukemia^29^, prolactinoma^30^, pancreatic cancer^31^ and NSCLC^32,33^. Thus, BIRC5 overexpression is associated with adverse outcome in various cancers including breast, lung, colorectal, prostate, and ovarian cancer^34^ and is considered to be one of the promising cancer diagnostic biomarker^35–38^. It has been reported that urine BIRC5 levels can serve as a diagnostic marker in case of bladder cancer^39^ and quantitative detection of BIRC5 in malignant pleural effusion can be useful in lung cancer diagnosis^40^. Recently, detection of serum BIRC5 levels has been found to be positively correlated with early diagnosis of different human cancers, including breast cancer^38^.

In this direction, present study was aimed to investigate the presence of BIRC5 protein in serum of dogs bearing CMT. At the same time, it was also necessary to estimate the levels of BIRC5 protein in healthy dogs and those suffering from non-cancerous disease ailments in order to evaluate the usefulness of BIRC5 protein as an additional marker associated with CMT in dogs. In this study, we report production, purification and characterization of dog rBIRC5 protein expressed in *E*. *coli* and development of a sensitive, label-free SPR biosensor assay using rBIRC5 specific IgGs immobilized sensor surface. This assay was used to detect serum BIRC5 protein in dogs suffering from CMTs. Serum BIRC5 levels were significantly higher in comparison to dogs with diseases other than CMT and healthy dogs. Thus, SPR immunosensor assay could be a useful tool to detect serum BIRC5 levels in dogs. To the best of our knowledge, this is the first report on real-time detection of serum BIRC5 levels in dog using a very sensitive, label-free SPR immunosensor.

## Materials and Methods

### Chemicals, buffers and reagents

*N*-(3-dimethylaminopropyl)-*N*-ethylcarbodiimide hydrochloride (EDC), *N*-hydroxy succinimide (NHS), Ethanolamine, Polyoxyethylenesorbitan monolaurate (Tween-20), Phosphate-buffered saline (PBS) (0.01Mphosphate, 0.138 M sodium chloride, 0.0027 M potassium chloride, pH 7.4), Sodium hydroxide (NaOH), hydrochloric acid (HCl), Lysozyme, Ampicillin, Sodium dodecyl sulphate (SDS), N,N-methylene-bisacrylamide, IPTG, skimmed milk powder, 3,3′-diaminobenzidine tetrahydrochloride (DAB), urea, glacial acetic acid, glycine, HRPO and FITC conjugates were purchased from Sigma-Aldrich. All chemicals were of either molecular biology grade or analytical grade and were used as received. All solutions were prepared in Type-II ultrapure water from ELIX Milli-Q system (Millipore).

### Tissue and serum samples

A total of 30 sera samples were collected from dogs surgically operated for removal of CMT mass. Along with this, 20 sera from dogs suffering from non-cancerous disease (NCD) conditions which were also presented to Referral Veterinary Polyclinic at Indian Veterinary Research Institute were included in this study. Further, sera from 20 healthy female dogs were used in the study as control animals. CMT tissues removed surgically were also collected in RNA*later* for extraction of mRNA to amplify, clone and express the target gene sequence. The tissues were also processed for histopathology and immunohistochemistry analysis and primary culture of canine mammary cells. This study was conducted as per guidelines and with due approval from the Institute Animal Ethics Committee (IAEC) of Indian Veterinary Research Institute, Izatnagar, India.

### RNA isolation and cDNA synthesis

Total RNA was extracted from surgically removed CMT tissue sample by RNeasy kit (Qiagen, Germany). Integrity of RNA sample was checked by running on 1.5% agarose gel and purity of RNA was checked by calculating ratio of OD_260_ and OD_280_ using biospectrometer (Eppendorf). Then cDNA was prepared from 1 µg total RNA using Revert Aid cDNA synthesis kit (Fermentas, USA) as per the manufacturer’s instructions in a total volume of 20 μl. The cDNA prepared was stored at −80 °C until used.

### Quantitative Real-time PCR

Expression of BIRC5 gene in tissue samples was determined by quantitative real time PCR. BIRC5 specific primers were used in the assay as reported by Jena and Co-workers^25^. The amount of input RNA was normalized using multiple endogenous controls namely β-Actin, 18sRNA, HPRT and RPS19. The reaction was carried out using KAPA SYBR FAST qPCR master mix (KAPA Biosystems, Boston, MA, USA) as per manufacturer’s instructions. Specificities of the PCR amplicons were confirmed by melt curve analysis. Ct values obtained from cDNAs from normal mammary gland tissue were used for calculating the relative gene expression levels in tumour cases using 2^−ΔΔCt^ method^41^. Data was expressed as mean values calculated from experiments performed in triplicate.

### Polymerase chain reaction (PCR) amplification and cloning of BIRC5 gene

Full length BIRC5 gene was amplified by PCR, using a set of primers, Surv FP (5′-TAG GAT CCA TCG GGT TTG AAT CGG G-3′) and Surv RP (5′-GGT AAG CTT CAG TGA TGG CAC GTT CT-3′) designed from available nucleotide sequence (accession number NM_001003348.1) of canine BIRC5 gene. Restriction sites for *BamHI* and *HindIII* (underlined in primer sequence) were introduced in the oligonucleotides to facilitate directional cloning. PCR was performed as 35 cycles at 98 °C for 20 s, 60 °C for 15 s and 72 °C for 15 s with 5 min initial denaturation and 7 min final extension using KAPA High Fidelity PCR master mix (2X) in a 50 μl reaction mixture containing 0.5 μl each of forward and reverse primer (20 pM) and 3 μl of template cDNA. The PCR amplified product was purified using mini elute PCR purification kit (Qiagen, Germany). The PCR product and vector pET-32b (+) were digested with *BamHI* and *HindIII* restriction enzymes. Then PCR product was ligated to pET-32b (+) vector using T4 DNA ligase (Promega, Madison, USA). The newly constructed recombinant plasmid was designated as pET-32b (+)-BIRC5 and was transformed into *E*. *coli* DH5α cells. Then positive clones were screened by colony PCR and further confirmed by RE digestion and sequencing. Thereafter, the pET-32b (+)-BIRC5 vector was transformed into BL-21 (DE3) *E*. *coli* competent cells.

### Induction of expression and purification of recombinant BIRC5 protein

*E*. *coli* harbouring pET-32b (+)-BIRC5 plasmid were grown in LB medium till OD_600_ nm reached 0.5–0.7. The cells were then induced with 1 mM IPTG and allowed to grow further for 6 h at 37 °C. Cells were harvested and proteins analyzed on 12% SDS-PAGE according to method given by Laemmli^42^. The recombinant protein was purified by Ni-NTA affinity chromatography using AKTA pure 25 M Fast Performance Liquid Chromatography (FPLC) (GE healthcare, Sweden) following manufacturer’s protocol. The culture pellet was solubilized with 8 M urea and lysate was loaded on HisTrap FF affinity column. Impurities were washed away with wash buffer containing 100 mM NaH_2_PO_4_, 10 mM tris-Cl and 8 M urea (pH 6.3) and finally eluted in elution buffer containing 100 mM NaH_2_PO_4_, 10 mM tris-Cl and 8 M urea (pH 4.5). Then recombinant protein was concentrated using the amicon ultra centrifuge filter (Millipore, Ireland) with 10 kDa membrane cut-off and further dialyzed in 1× PBS to remove urea. The concentration of purified protein was determined by Qubit™ protein assay kit (Invitrogen, USA) according to the manufacturer’s protocol.

### Characterization of rBIRC5 protein by western blotting

To confirm the immunoreactivity and specificity of dog rBIRC5 protein, immunoblot analysis was performed. A 3 µg aliquot of purified dog rBIRC5 protein was separated on 12% SDS-PAGE and electrophoretically transferred onto a Nitrocellulose membrane. After blocking with 5% skimmed milk overnight at 4 °C, the membrane was incubated for 2 hr with rabbit anti-human BIRC5 primary antibody (Santa cruz biotechnology Inc, California, USA; Cat # sc-10811) diluted to 1:150 in PBS. The membrane was subsequently incubated with goat anti-rabbit horse radish peroxidase (HRPO) conjugate (Sigma-Aldrich, U.S.A; Cat # A3687) diluted to 1:3000 with PBS, for 1 hr at 37 °C. After washing with PBST, antigen-antibody complexes were developed using diaminobenzidine (DAB) and developed blot was photographed by gel doc XR+ imager (Bio-Rad, USA).

### Generation of polyclonal antibodies in guinea pigs

Two guinea pigs weighing about 500 g each, obtained from Laboratory Animal Resources (LAR), Facility at IVRI, Bareilly, India were injected subcutaneously with purified rBIRC5 protein @300 μg per animal after collecting pre-immune blood samples from each animal. For immunization, first dose of rBIRC5 protein was given as a 1:1 emulsion with Freund’s complete adjuvant (FCA); whereas for subsequent boosters, Freund’s incomplete adjuvant (FIA) was used. Immunization was performed at weekly interval and four doses were administered to obtain the hyper immune serum (HIS). Ten days after the final injection, blood samples were collected by cardiac puncture and sera were kept at −80 °C in aliquots until used.

### Purification of protein specific IgGs from the hyper immune sera

IgGs were purified from HIS raised against rBIRC5 protein using ‘Melon Gel IgG Purification kit’ (Thermo Scientific, USA) as per manufacturer’s instructions. Purified IgG were collected in a microfuge tube and protein concentration was estimated before storage at −80 °C. For purification of protein specific IgGs, the rBIRC5 protein was treated with EnterokinaseMax kit (Invitrogen, USA; Cat # E-180-01), which contains enzyme enterokinase, that recognizes a sequence -(Asp)4 Lys and cleaves after lysine residue to remove the histidine tag from recombinant protein containing fusion tag. Thereafter, 1 mg of purified rBIRC5 protein without histidine tag was dissolved in 1.5 ml coupling buffer (0.1 M sodium phosphate, 0.15 M NaCl, pH 7.2), and added to 100 mg NHS-activated agarose dry resin (Pierce, Thermo scientific, USA) in a spin column and kept for shaking initially at room temperature for 1 hr and then at 4 °C for overnight. The column was washed thrice with 2 ml of coupling buffer. Then 1 M ethanolamine was added and the column was again rotated for 20 min at RT to block the unbound sites on the resin. The column was further washed thrice with 2 ml coupling buffer and then equilibrated with binding buffer (PBS, pH 7.4). The purified IgGs obtained from 1 ml HIS were diluted 1:1 (v/v) in PBS and added to protein bound agarose resin in spin column. The column was kept overnight at 4 °C in a tube rotator to mix resin and buffer containing IgGs. Then column was washed with 3 ml binding buffer and finally protein specific IgGs were eluted using 0.1 M glycine, pH 2.8. The purified IgGs were dialyzed in PBS and stored at 4 °C till further use. The concentration (mg/ml) of purified rBIRC5 protein IgGs was calculated using the formula: A_280nm_/Absorbance coefficient of guinea pig IgG. Different steps involved in immunosensor based detection of BIRC5 in dog serum are shown diagrammatically in Fig. S1.

### Characterization of antibodies for reactivity with native BIRC5 protein

#### Immunohistochemistry

Tumour tissues fixed in 10% normal buffered formalin were processed, paraffin embedded and cut into 4–5 µm thick sections which were taken on positively charged poly L-Lysine coated microscopic slides. The slides were kept in hot air oven at 60 °C for 1 hour for melting paraffin and allowing proper spread of sections. After this, sections were rehydrated immediately by dipping in xylene solution to remove paraffin by two changes of xylene (5 minutes each), followed by descending grades of alcohol (Alcohol I, II, 90%, 80%, 70%, 50% for 3 minutes each). Then washed with distilled water in coplin jar for 5 minutes. Antigen retrieval was performed by heat induced epitope retrieval method to expose the masked epitopes. The slides were then incubated in 3% H_2_O_2_ in absolute methanol for 30–45 minutes at room temperature in dark for quenching/blocking endogenous peroxidise followed by thorough washing in PBS. Blocking was done further using 5% dog serum in PBS (pH 7.4) at room temperature for 30–45 min. Slides were then incubated with 1:100 dilution of purified IgGs followed by three rinses in PBS and addition of anti-guinea pig horse radish peroxidase conjugated secondary antibody in 1:50 dilution to cover the moist sections. After thorough rinsing in PBS, 3,3′-diaminobenzidine (DAB) substrate was added for 3 min. The reaction was terminated by washing slides in distilled water. The slides were further counter-stained with Mayer’s hematoxylin for 30 sec then washed thoroughly with water. Immunohistochemical section was dehydrated in ascending grades of ethanol starting from 50% to absolute ethanol for 5 minutes each. Final clearing was done in xylene and tissue was preserved by mounting in DPX mountant.

#### Immunocytochemistry

For immunocytochemistry, cells from REM-134 canine mammary cancer cell line and primary canine mammary tumour cells cultured from a clinical case of CMT were used. The cells were seeded on a sterile glass cover slip kept in 4 well plates and incubated at 37 °C in a CO_2_ incubator in presence of 5% CO_2_. After reaching confluency, the cells were washed thrice in 1X Dulbecco’s phosphate buffered saline (DPBS) to remove dead and loosely adhered cells. The cells were fixed in 80% acetone for 10 min at room temperature and then washed thrice in 1X DPBS. Permeabilization was done with Triton-X (0.2% in PBS) for 10 min and then washed thrice in 1X DPBS. Blocking was done in 2% BSA at 37 °C for 1–2 hour and then again washed thrice in 1X DPBS. After blocking, 200 μl of 1:100 dilution of purified IgGs was added to react with BIRC5 protein. The cells were incubated overnight at 4 °C and then washed 3 times with PBS. The plate was again kept in incubator at 37 °C for 2 hours with 200 μl of 1:200 diluted anti-guinea pig FITC conjugate. DAPI was used as nuclear stain. The cells were washed 5 times with PBS and then visualised under fluorescence microscope (Olympus BX53).

### SPR biosensor system

Real-time, label-free biomolecular interactions between purified IgGs and BIRC5 protein were recorded using a dual-channel kinetic evaluation instrument (KEI) ESPRIT Twingle Biosensor platform (KEI, Netherlands). Bare gold SPR sensor discs with a 50 nm thick coating of gold on glass surface were supplied along with equipment. The system is equipped with a single laser light source of 670 nm and a diode detector. This is an open cuvette based, dual channel system in which channel-1 was used to measure interactions between BIRC5 protein and antibodies and channel-2 was used as a reference to monitor signals due to change in refractive index of the buffers. Different reagents, samples and buffers were injected in desired amount in two cuvettes (assembled over gold disc). The buffers from the cuvettes were drained by two peristaltic pumps which were also used for washing the chip surface after every step. The flow of running buffer (10 mM PBS containing 0.05% Tween-20, pH 7.4) was software controlled and programmed to stop during each incubation step. All experiments were performed at 25 °C and temperature around cuvette and samples were maintained by circulating the water at desired temperature using a water bath circulator (Julabo F-32, Germany) connected with the system. This equipment is based on Kretscmann configuration, the most commonly used optics in majority of SPR-based systems, which involves measuring excitation of surface plasmons at planar surface using a light source. This technique is used extensively to characterize binding interactions between any two molecules without any labelling requirements. Interaction between surface-bound IgGs and BIRC5 protein present in test samples were recorded as a differential of response in channel-1 and channel-2, thereby deducting the contribution of running buffer (PBS, pH 7.4) in each run. The immunosensor response was acquired using data acquisition software and analyzed using kinetic evaluation software version 5.4 provided with system.

### Covalent immobilization of IgG on SPR sensor disc

Bare gold SPR sensor disc was cleaned with absolute ethanol and dried in a stream of nitrogen gas. Thereafter, self-assembled monolayer (SAM) of 11-mercaptoundecanoic acid (MUA) was created by treatment with 1 mM MUA in ethanol for 12 hours. The –SH groups of MUA reacts with gold surface to create a stable disulphide bond resulting in generation of COOH groups on gold sensor surface. The unreacted MUA was removed by washing chip with ethanol four to five times and finally washing with deionized water. The chip was dried in a stream of nitrogen and then docked on the SPR biosensor system for further experiments.

**Figure 1:**
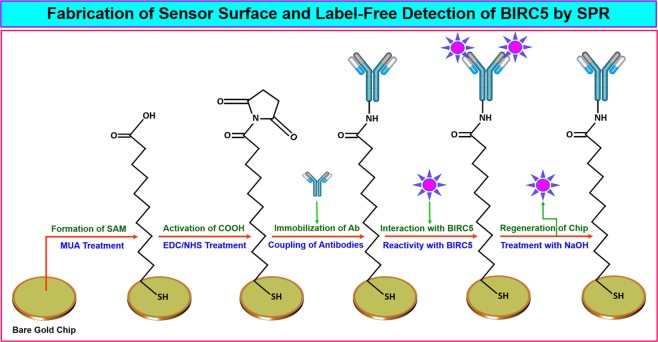
Schematic representation of fabrication of bare gold SPR sensor surface and detection of BIRC5. The steps involved are formation of SAM of MUA on bare gold surface, activation of COOH groups with EDC/NHS, covalent coupling of antibodies on activated surface, detection of BIRC5 present in serum and treatment of chip with NaOH to regenerate surface for next round of interaction.

Before immobilization of IgGs, the surface of SAM modified chip was cleaned using coupling buffer (10 mM sodium acetate buffer) till a stable baseline was obtained. The ligand (IgGs) was covalently immobilized on surface using amine coupling strategy, exploiting primary amine groups on IgGs. Initially, the IgGs were diluted to a concentration of 150 µg/ml in 10 mM sodium acetate buffers (pH 4.0, 4.5, 5.0 and 5.5) and 50 µl of each solution was injected individually on the sensor surface to select the optimum buffer pH which showed highest signals during 60 s run. After each run, 50 µl of 100 mM NaOH was used to clean the chip surface. Then sensor surface was stabilized by passing coupling buffer (acetate buffer, pH 4.5 which was found optimum in this case) repeatedly over sensor chip in both the channels. For covalent coupling of IgGs, 0.4 M EDC and 0.1 M NHS were prepared fresh in filtered deionized water and COOH groups present on sensor surface in both the channels were activated by passing a mixture (1:1 v/v) of EDC and NHS for a period of 300 s. The IgGs dissolved in coupling buffer @150 µg/ml was immobilized in channel-1, whereas, only coupling buffer was passed in channel-2 for 600 s. After immobilization, remaining activated COOH groups in both the channels were blocked with 1 M ethanolamine (pH 8.5) for a period of 600 s and chip surface was washed twice with running buffer. Preparation of SPR sensor surface was monitored real-time using data acquisition software supplied with the system. The chemistry used for preparation of sensor surface and interaction with BIRC5 is explained in the Fig. 1.

### Determining limit of detection (LoD) of assay and interaction of BIRC5 protein present in sera with IgGs immobilized on chip

Interactions of IgGs immobilized on sensor chip with two-fold serially diluted rBIRC5 protein (400 pg/ml to 3.125 pg/ml) in running buffer were recorded in duplicate for each dilution and shift in millidegrees were recorded to determine the LoD of assay. The calibration curve was obtained by plotting response values for each dilution in MedCalc 9.2 software. Thereafter, sensorgrams for interaction between chip bound IgGs and BIRC5 protein present in the sera of dogs suffering from CMT, other non-cancerous diseases, as well as, healthy serum samples were recorded in real-time and analyzed with kinetic evaluation software version 5.4. The serum samples were diluted 1:20 in running buffer and 50 µl of each sample was injected in channel-1 over immobilized IgGs and 50 µl of running buffer was injected in channel-2. The binding interactions were recorded for 300 s (association). Loosely bound antibodies were removed by passing running buffer for 90 s (dissociation). The sensor surface was regenerated with 50 mM NaOH. All experiments were performed in duplicate at 25 °C and data is presented as averages of the two runs for each sample.

### Sandwich ELISA for detecting BIRC5 protein

BIRC5 protein was detected in dog serum samples using canine survivin (SURV) ELISA Kit (Cat No. MBS022408) procured from MyBiosource as per manufacturer’s instructions. Briefly, all reagents and serum samples were brought to room temperature. Thereafter, 50ul of standards and each test sample, as well as, 50 ul of sample diluent is added to pre-coated plate wells. The HRP conjugate is also added @100ul/well and incubated at 37 °C for 60 min. After washing the plates four times with washing buffer, chromogenic substrate is added @50ul per well and incubated at 37 °C for 15 min. Thereafter, stopping solution was added @50ul per well, optical density at 450 nm was measured for each well and calculations were made as per manufacturer’s instructions.

### Statistical analysis

To determine the presence of BIRC5 protein in test serum samples, SPR response was averaged from duplicate experiments. ROC curves, area under curve (AUC) and sensitivity and specificity were determined with MedCalc 9.2 software. The method of DeLong and Co-workers^43^ was used for calculation of standard error of AUC and an exact binomial confidence interval for AUC was calculated. Statistical analysis of SPR data was done using Prism 5.0d (GraphPad Software Inc., La Jolla, CA, USA) software and statistical differences between groups were determined using one-way analysis of variance (ANOVA) followed by Tukey’s post-hoc tests for comparison between groups.

### Ethical approval

All experimental procedures involving use of laboratory animals were in accordance with Breeding of and Experiments on Animals (Control and Supervision) Amendment Rules, 2005. The experiments were conducted with proper permission from the Institutional Animal Ethics Committee (IAEC), Indian Veterinary Research Institute, Izatnagar and the Committee for the Purpose of Control and Supervision of Experiments on Animals (CPCSEA), Ministry of environment and forests.

## Results

The entire procedure and experimental plan for developing label-free SPR biosensor assay to detect BIRC5 protein in dog sera using purified anti-BIRC5 antibodies immobilized on sensor chip is presented as layout in Fig. S2 in supplementary information.

### Over-expression of BIRC5 gene in canine mammary tumours

Overexpression of BIRC5 mRNA transcripts was confirmed in CMT tissues by real time PCR. Relative expression levels of BIRC5 gene were 5.6 ± 0.462 to 60.0 ± 1.476 fold higher in the CMT tissue samples as compared to normal healthy mammary gland tissues. More than five-fold higher expression of BIRC5 gene was detected in 12 out of 16 (75%) CMT tissues examined in this study (Fig. S3 in supplementary information). The results of real-time PCR study were further validated by IHC of tissues showing BIRC5 gene expression. Positive correlation obtained between real-time PCR and IHC studies (r = 0.89), confirmed overexpression of BIRC5 in CMT tissues.

### Amplification and cloning of BIRC5 gene

Full length coding sequence of BIRC5 (516 bp) was amplified from a confirmed case of dog complex mammary carcinoma showing about 60 fold overexpression of BIRC5 gene (by quantitative real-time PCR analysis) as compared to normal mammary gland tissue^25^ and cloned in pET32b(+) prokaryotic expression vector as described in materials and methods. The recombinant clones were confirmed by PCR, restriction endonuclease analysis and plasmid DNA sequencing. Sequencing data revealed correct orientation of the BIRC5 gene in pET32b (+) vector. BIRC5 sequence was submitted to GenBank (Accession No. KT693114) and it showed >90% similarity with the BIRC5 gene from other species [*Felis catis* (93%), *Equus cabalus* (92%), *Homo sapiens* (91%), *Bos taurus* (90%), *Bubalus bubaus* (90%), *Ovis aries* (90%) *Capra hircus* (90%)], thus revealing conserved nature of this gene^25^.

### Expression, purification and characterization of dog BIRC5 protein

The recombinant plasmid (pET32b-BIRC5) was transformed in *E*. *coli* BL21 (DE3) cells and expression of rBIRC5 protein was induced with 1 mM IPTG. Upon 12% SDS PAGE analysis, cell lysates from induced cultures showed intense 37 kDa band corresponding to recombinant BIRC5 protein. The recombinant protein was purified by Histrap FF column (GE Healthcare) and eluted histidine tagged protein showed a single band of 37 kDa molecular weight in SDS-PAGE analysis (Fig. S4B, supplementary information). The yield of recombinant protein was found to be around 2 mg per 100 ml of induced bacterial culture. Recombinant protein was confirmed as BIRC5 upon immunoblot analysis with anti-human BIRC5 antibodies which showed colour development at position corresponding to 37 kDa recombinant BIRC5 protein as shown in Fig. S4A.

#### Characterization of anti-BIRC5 antibodies by Immuno-histochemistry and Immuno-cytochemistry

**Figure 2:**
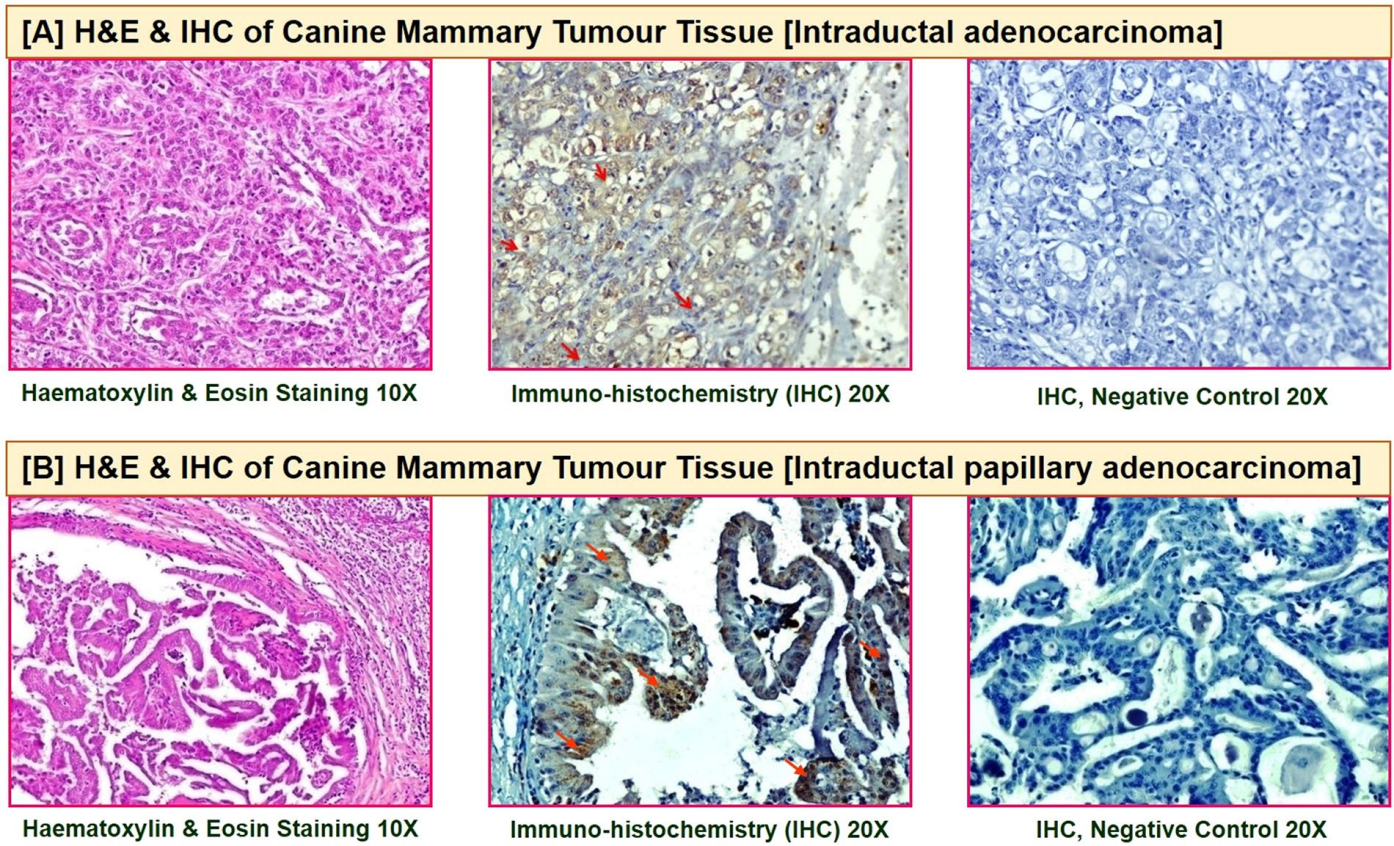
Immunohistochemistry for characterization of anti-BIRC5 antibodies: The purified antibodies reacted specifically with the native BIRC5 protein in CMT tissues (**A**,**B**).

**Figure 3:**
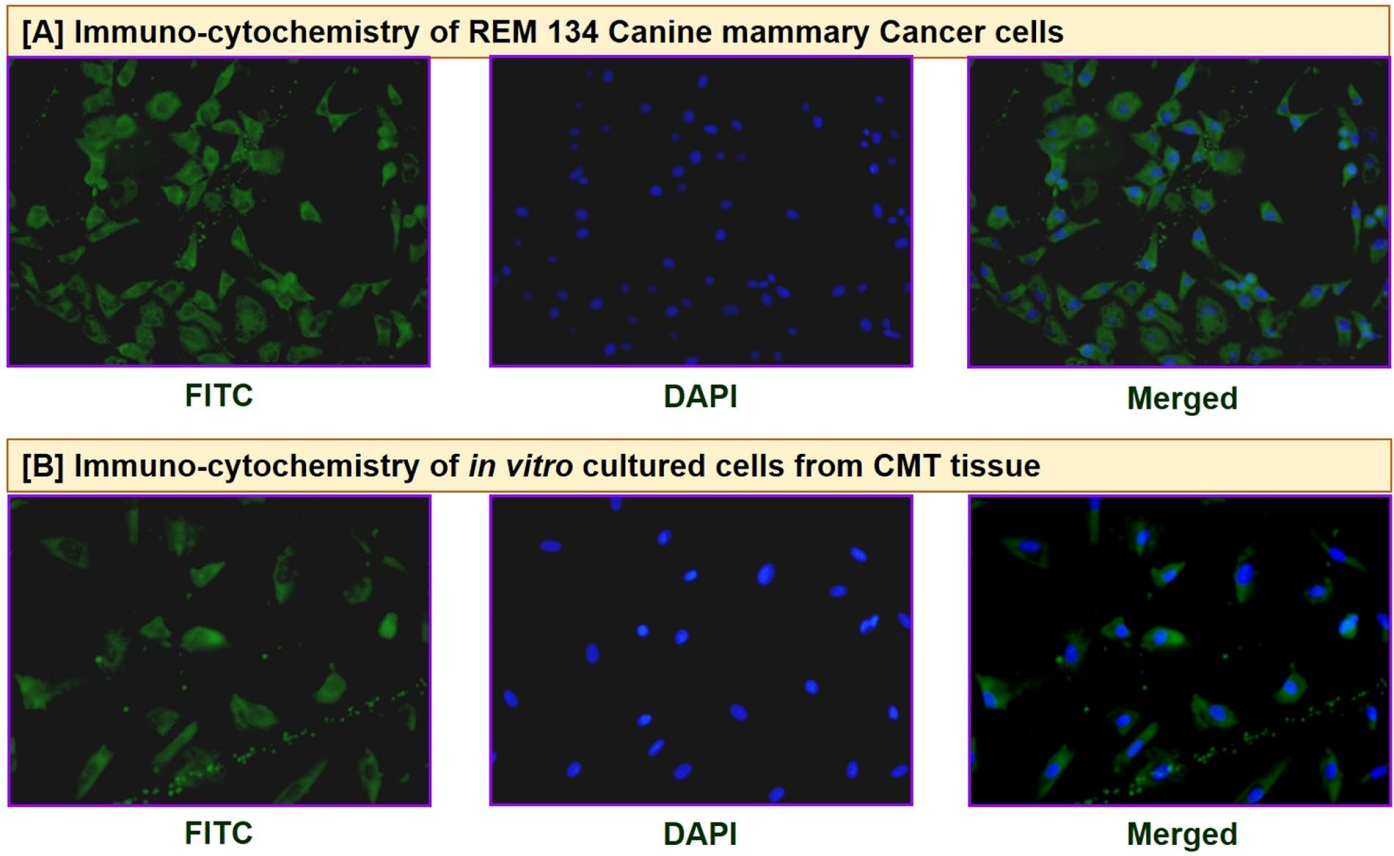
Immunocytochemistry for characterization of anti-BIRC5 antibodies: The purified antibodies reacted specifically with the native BIRC5 protein in canine mammary cancer cells (**A**,**B**).

Purified IgGs raised in guinea pig against recombinant BIRC5 protein were characterized by IHC for detection of native BIRC5 in CMT tissues. The results indicate that antibodies reacted specifically with native BIRC5 in tissues resulting in intense cytoplasmic staining as shown in Fig. 2(A,B), however, no signal was observed in control cells obtained from normal mammary gland tissues. Similarly, presence of BIRC5 protein in primary culture of canine mammary tumour cells, as well as, REM-134 canine mammary cancer cell line was determined by immuno-cytochemistry. The cells showed specific signals upon immunofluorescence staining using purified anti-BIRC5 antibodies and FITC conjugated anti-guinea pig antibodies Fig. 3(A,B). These antibodies were then utilized to develop a label-free immunosensor assay for detecting serum BIRC5 in dogs.

### Optimizing conditions for immobilization of IgGs on sensor surface

Based on shift in SPR dip, which was recorded for ligand dissolved in acetate buffer of different pH (4.0 to 5.5); the acetate buffer (pH 4.5) was selected to covalently couple IgGs on sensor chip. The COOH groups available on SAM modified sensor chip were activated with a mixture of EDC/NHS and ester groups thus generated were covalently coupled with NH_2_ present on antibody ligand to form stable amide bonds. The unreacted COOH groups on sensor chip were blocked by treatment with ethanolamine (pH 8.5). In channel-2, all activated COOH groups were blocked with ethanolamine to serve as reference control. The immobilization of antibodies on sensor chip was monitored with data acquisition software.

SPR response during immobilization of purified IgGs on sensor surface can be converted to mass loading according to relationship: 122 m° shift = 1 ng/mm^2^ of immobilized ligand. Accordingly, a net change in angle (Δθ) of 424.54 m° is equivalent to covalent attachment of 3.54 ng/mm^2^ of purified IgG on sensor surface (Fig. S5).

### Determining limit of detection (LoD) of the assay and affinity kinetics

**Figure 4:**
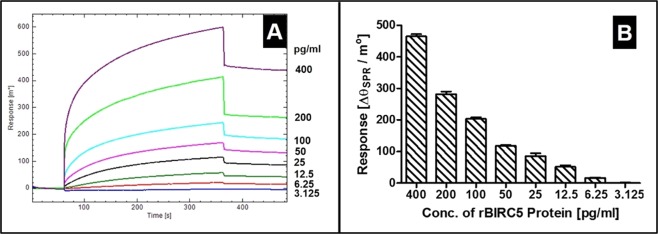
Overlay of SPR sensorgrams (**A**) and Bar graph (**B**) showing interaction of different concentrations of recombinant BIRC5 protein (400 pg/ml to 3.125 pg/ml) with protein specific antibodies immobilized on sensor chip.

To determine LoD of assay, a twofold dilution series of rBIRC5 protein was reacted with IgGs immobilized on sensor surface. Upon decrease in concentration of rBIRC5 from 400 pg/ml to 3.125 pg/ml, a proportionate shift in SPR response from 458.554 m° (0.0032 RIU) to 0.710 m° (−4.99E-06 RIU) was observed (presented in Fig. 4A) indicating an effective interaction between IgG and BIRC5 protein. SPR response was recorded after completion of dissociation phase (at 480 sec) for each dilution in duplicates and average values are plotted as bar diagram (Fig. 4B). However for clarity of presentation, overlay of only one sensorgram for each dilution is presented in Fig. 4A. The data clearly indicated strong interaction between BIRC5 protein and IgGs.

**Figure 5:**
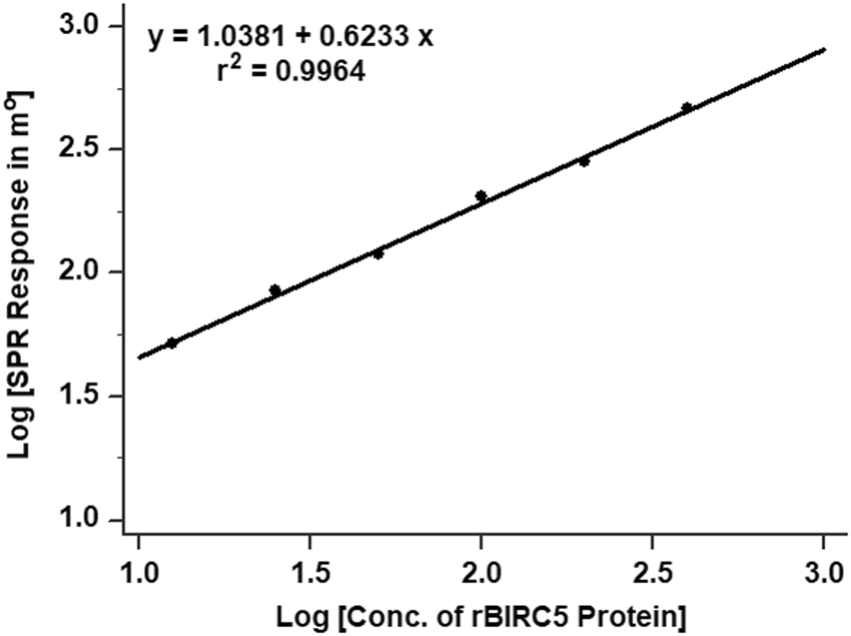
Calibration curve showing goodness of fit between SPR responses obtained upon interaction of different concentrations of recombinant BIRC5 protein with specific antibodies immobilized on sensor surface. The regression equation y = 1.0381 + 0.6233 x and r^2^ = 0.9964 indicates that the regression model fits data much better than the null hypothesis.

A calibration curve plotted to determine LoD of assay is presented in Fig. 5, which shows goodness of fit between SPR response obtained upon interaction of different concentrations of BIRC5 protein with specific antibodies immobilized on sensor surface. The regression equation y = 1.0381 + 0.6233x and r^2^ = 0.9964 indicates that regression model fits data much better than null hypothesis and SPR response obtained for different concentrations of BIRC5 protein followed a linear trend within a range 400 to 6.25 pg/ml. Therefore, the detection limit of SPR assay was 6.25 pg/ml, which is lower than average levels of BIRC5 protein estimated in healthy dog sera (19.88 ± 1.861 pg/ml) analyzed in this study and also below the detection limits of reported enzyme-labeled immunoassays^44,45^.

The affinity interactions were determined by numerical fitting of data in a single 1:1 monophasic model (A + B ↔ AB) wherein the concentration of analyte is assumed to be homogenous throughout solution and remained constant during the association phase. The overlay of curves for different concentrations of BIRC5 protein were fitted into a numerical model using a global analysis technique. Since binding constants were same in all interaction plots and only the concentration of protein was different, therefore all plots were fitted at the same time to same set of parameter values to ensure that selected model effectively fits to experimental data. The association and dissociation rate constants were determined as 1.789 × 10^9^ M^−1^s^−1^ and 2.18 × 10^−2^ s^−1^, respectively. The equilibrium dissociation constant, (K_D_ = k_d_/k_a_) was 12.1 × 10^−12^ M; which indicates that antibodies are of high affinity with sensitivity in picomolar range. The reproducibility of SPR assay was determined by running two different samples on sensor chip at five occasions. The sensor surface was regenerated using regeneration buffer (50 mM NaOH) each time before next sample was analyzed. As shown in Fig. S6, we obtained reproducible results for both the samples tested and there was near perfect super-imposition of the interaction curves. This indicated that the antibody coated chip was robust and the concentration of regeneration buffer was perfect for regenerating the chip after every run without loss of ligand from the chip.

### Specificity and sensitivity of SPR assay for detecting BIRC5 protein in dog sera

The diagnostic sensitivity and specificity of SPR immunosensor was determined by testing 70 dog sera samples, including samples from confirmed cases of CMT (n = 30); sera from dogs suffering with non-cancerous disease conditions (n = 20) and sera from healthy female dogs (n = 20). Each serum sample was tested in duplicate, sensograms were recorded and average values were obtained at the end of dissociation phase.

**Figure 6:**
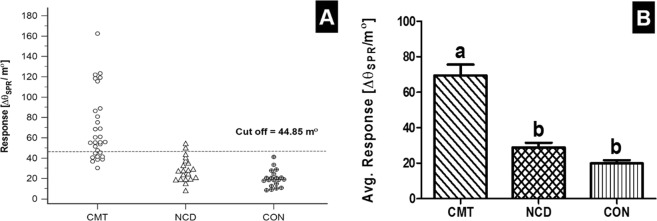
SPR response of dog sera samples collected from clinical cases of canine mammary tumour (CMT), diseases other than cancer (NCD) and healthy dogs (CON). (**A**) Dot plot showing distribution of samples. (**B**) Bar diagram showing mean SPR response [m°]. Total 70 dog sera samples including 30 from canine mammary tumour (CMT), 20 from dogs with non-cancerous disease (NCD) conditions and 20 healthy (CON) dogs were tested for the presence of BIRC5 protein by labeI-free SPR biosensor assay. The cut-off limit for the assay was taken as 44.85 m°, which was defined as SPR response value greater than twice standard deviation above the mean response of the controls. The mean SPR response in the CMT cases differ significantly with the healthy control and non-cancerous diseased dogs (p < 0.001). In Fig. 6B, groups with different alphabetical superscripts differ significantly (p < 0.001).

The average SPR response (Mean ± SEM) of sera samples from 20 healthy dogs was found to be 19.88 ± 1.86; which is equivalent to 30.23 ± 1.32 pg/ml of BIRC5 protein. The positive/negative cut-off limit of this assay was taken as 44.85 m°, that was defined as response value greater than twice SDs above mean value of healthy dogs (Fig. 6A). The frequency of BIRC5 positive sera in cancerous dogs was significantly higher (p < 0.001) than dogs with non-cancerous diseases and healthy dogs. The mean SPR response ± SEM values in dogs with CMT and NCD were 69.50 ± 6.08 and 28.91 ± 2.66; and equivalent concentration of BIRC5 protein was 109.83 ± 8.10 pg/ml and 44.71 ± 2.61 pg/ml, respectively. The mean SPR response in CMT dog sera was significantly higher (p < 0.001) than healthy and non-cancerous diseased dogs (Fig. 6B).

Although SPR response was greater than cutoff limit of assay in two dogs suffering from pneumonia and gastroenteritis (NCD), no significant difference was observed between sera samples from NCD and healthy dogs (p < 0.001). The average values (Mean ± SEM) of different binding parameters namely SPR response, BIRC5 concentration, surface density, effective shift in RIU, amount and number of BIRC5 protein molecules bound on sensor surface for all sera samples analyzed in different groups (CMT, NCD and control) are presented as bar diagrams in the Fig. S7(A–D) of supplementary infromation, respectively.

**Figure 7:**
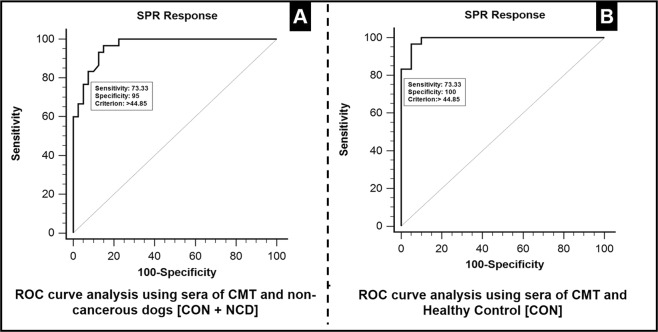
Diagnostic performance of SPR biosensor assay: (**A**) ROC Curve analysis showing SPR response of sera samples from cases of CMT and non-cancerous dogs including those with non-cancerous diseases (NCD). The diagnostic performance of SPR biosensor assay to discriminate sera samples collected from dogs with CMT and non-cancerous dogs (including NCD and control) was evaluated by ROC curve analysis. Each point on the ROC curve represent a sensitivity/specificity pair corresponding to a particular decision threshold. The area under curve (AUC) was 0.964 and 95% confidence interval of the AUC for SPR assay ranged from 54.1–87.7. At cut-off value of 44.85 m°, the specificity and sensitivity of assay were 95% and 73.33% respectively. The AUC indicated that SPR biosensor assay has an ability to distinguish between the two groups. Similarly, figure (**B**) shows ROC Curve analysis for SPR response of sera samples from CMT and healthy dogs. At cut-off value of 44.85 m°, the specificity and sensitivity of assay were found to be 100% and 73.33% respectively.

The diagnostic performance of SPR biosensor assay to discriminate sera samples collected from dogs with CMT and non-cancerous dogs (including NCD and control) was evaluated by ROC curve analysis. Each point on the ROC curve represented a sensitivity/specificity pair corresponding to a particular decision threshold. The area under curve (AUC) was 0.964 and 95% confidence interval of AUC for SPR assay ranged from 54.1–87.7. At cut-off value of 44.85 m°, the specificity and sensitivity of assay were 95% and 73.33% respectively (Fig. 7A and Table 1).

**Table 1:** ROC curve analysis of SPR biosensor assay between different groups of sera samples collected from dogs with CMT, NCD conditions and healthy dogs.

	AUC	95% CI	Sensitivity	Specificity	SE	Association Criterion
CMT Versus NCD + Control	0.964	54.1–87.7	73.33	95.00	0.0180	>44.85
CMT Versus Control	0.990	54.1–87.7	73.33	100.00	0.0095	>44.85

The AUC indicated that SPR biosensor assay has an ability to clearly distinguish between two groups. Further ROC curve analysis of sera samples from cases of CMT and healthy (control) dogs showed specificity and sensitivity as 100% and 73.33% respectively (Fig. 7B and Table 1).

As negative control of SPR assay, representative CMT, NCD and CON samples were reacted with unrelated antibodies (raised in guinea pig against synthetic peptide antigen corresponding to viral protein) immobilized on SPR sensor surface (Fig. S8). These samples showed negligible SPR signals (Fig. S9), thereby demonstrating specificity of the immunosensor assay.

### Comparative evaluation of SPR and Sandwich ELISA for detecting BIRC5 protein in dog sera

When all serum samples were tested in parallel using SPR and commercially available canine survivin ELISA kit, the sensitivity and specificity of SPR assay was 73.33% and 95% respectively, whereas that of sandwich ELISA was 66.67% and 92.50% respectively. The detection range claimed for this sandwich ELISA kit is between 15.6 to 500 pg/ml whereas, in SPR assay the detection limit was in the range 6.25 to 400 pg/ml, thus detecting lower levels of BIRC5 in the test samples. The interaction time was optimized to 480Sec for each sample run and the interactions between two molecules were monitored in real-time. In case of sandwich ELISA, 100ul serum sample was required to test a sample in duplicate whereas, in case of SPR assay, only 10ul of sample is needed to obtain results in duplicate. In case of SPR, same antibody fabricated surface was regenerated after each cycle of interaction thus economizing the cost per sample. Since the instrument is fully automated, it can analyse multiple samples in an unattended run, which further reduces cost and manual intervention unlike ELISA where reagents like capture and detection antibodies, conjugate and substrate are used for analysis of each sample, which involves costs and labor. Thus SPR immunosensor assay developed in this study is useful for screening large number of dog sera samples.

## Discussion

In case of cancers, both auto-antibodies and proteins secreted in the body fluids, act as biomarkers; and many groups have developed different biosensor techniques to identify serum cancer biomarkers^46–49^. These include, both label-free techniques and other assays using different detection labels like enzymes and fluorophores. The SPR has been used extensively to study antigen/antibody interactions and measure their binding kinetics^50,51^. In majority of diagnostic interventions, this technique has been utilized to detect presence of antibodies in serum using an immobilized antigen ligand on the chip^52^. SPR assays has been reported for detecting protein biomarkers in human sera in cases of pancreatic cancer^46^, ovarian cancer^49^ and coronary artery disease^24^. However, majority of studies which report detection of BIRC5 in dog cancers such as CMT^53^, nasal carcinoma^54^, cutaneous squamous cell carcinomas^55^, canine cutaneous sebaceous tumour^56^, canine osteosarcomas^57^, canine mast cell tumours^58^ etc. are based on immunohistochemistry analysis using specific antibodies. Similarly, autoantibodies to BIRC5 protein in dog sera has been reported earlier^25,26^; but a rapid and sensitive assay for detecting BIRC5 protein in dog sera is not yet reported and to the best our knowledge, this is the first report on application of SPR biosensor technique for detecting BIRC5 protein biomarker in dog sera.

Serum BIRC5 levels in different cases of human breast cancer has been quantitated using ELISA^44^. It was reported that values <50 pg/ml were considered normal while a BIRC5 level of 50–70 pg/ml was considered mild, 70–90 pg/ml as moderate and >90 pg/ml as high. It was also concluded that chemotherapy/treatment may affect patient serum BIRC5 levels. In our study, we found that serum BIRC5 levels were 109.83 ± 8.10 pg/ml in dogs with CMT as compared to 44.71 ± 2.61 pg/ml and 30.23 ± 1.32 pg/ml respectively, in case of dogs with non-cancerous diseases and healthy subjects. In a recent study, Goricar *et al*.^59^ has reported that detection of serum BIRC5 levels before and during chemotherapy could serve as a biomarker for predicting malignant mesothelioma treatment response in humans. Thus, the SPR immunosensor based detection of BIRC5 protein in sera may serve as a prognostic biomarker if it is conclusively proved that BIRC5 protein starts appearing in serum before the tumour mass is apparently visible. A systematic study in experimentally induced CMT may unravel this hypothesis and under such conditions, the immunosensor developed using BIRC5 protein specific antibodies in this study, could prove to be a useful prognostic assay.

## Conclusion

In case of CMT, over expression of BIRC5 gene results in increased secretion of protein in body fluids, which acts as biomarker and serve as diagnostic target. Development of sensors for identifying and detecting serum cancer biomarkers is an emerging area. In this direction, efforts were made to develop a rapid, label-free immunosensor assay using SPR technique to detect BIRC5 protein and discriminate dogs with CMT from healthy and non-cancerous disease counterparts. High binding affinity due to strong interaction between BIRC5 and antibodies resulted in increased specificity and sensitivity with LoD of 6.25 pg/ml of assay. These findings suggest that SPR immunosensor based detection of BIRC5 in dog sera could be used as a sensitive tool for CMT diagnosis. However, to confirm its utility for early diagnosis and prognosis, systematic study in experimentally induced CMT in dogs need to be taken up for monitoring serum BIRC5 levels, before onset of tumour, during tumour progression and after surgical removal of tumour mass or chemotherapy. Since SPR biosensor system is a fully automated platform for sample and reagent handling, the immunosensor assay can be successfully used for screening large number of samples in an unattended run. Further work can be taken up for developing ‘on-site’ sensors by optimizing the assay conditions using these tested reagents on some portable biosensor platform, which may provide near-patient testing of samples in clinical settings.

## Supplementary information

Surface plasmon resonance immunosensor for label-free detection of BIRC5 biomarker in spontaneously occurring canine mammary tumours

## Data Availability

All relevant data pertaining to this study has been presented in the paper and as supplementary information.
