# Supplementary material for: Surface plasmon resonance immunosensor for label-free detection of BIRC5 biomarker in spontaneously occurring canine mammary tumours
Source: Sci Rep. 2019 Sep 17;9:13485. doi: 10.1038/s41598-019-49998-x (PMC6748992; doi:10.1038/s41598-019-49998-x)
Supplement: Supplementary file 1 — Surface plasmon resonance immunosensor for label-free detection of BIRC5 biomarker in spontaneously occurring canine mammary tumours [file 41598_2019_49998_MOESM1_ESM.docx]

**Surface plasmon resonance immunosensor for label-free detection of BIRC5 biomarker in spontaneously occurring canine mammary tumours**

Subas Chandra Jena^1#^, Sameer Shrivastava^1*#^, Sonal Saxena^1#^, Naveen Kumar^2^, Swapan Kumar Maiti^2^, Bishnu Prasad Mishra^1^ and Raj Kumar Singh^1^

**^1^Facility for Research and Training on Bioassays and Biosensor, Division of Veterinary Biotechnology, ^2^Division of Surgery, ICAR-Indian Veterinary Research Institute [Deemed University] Izatnagar, 243122, India**

**Supplementary Information**


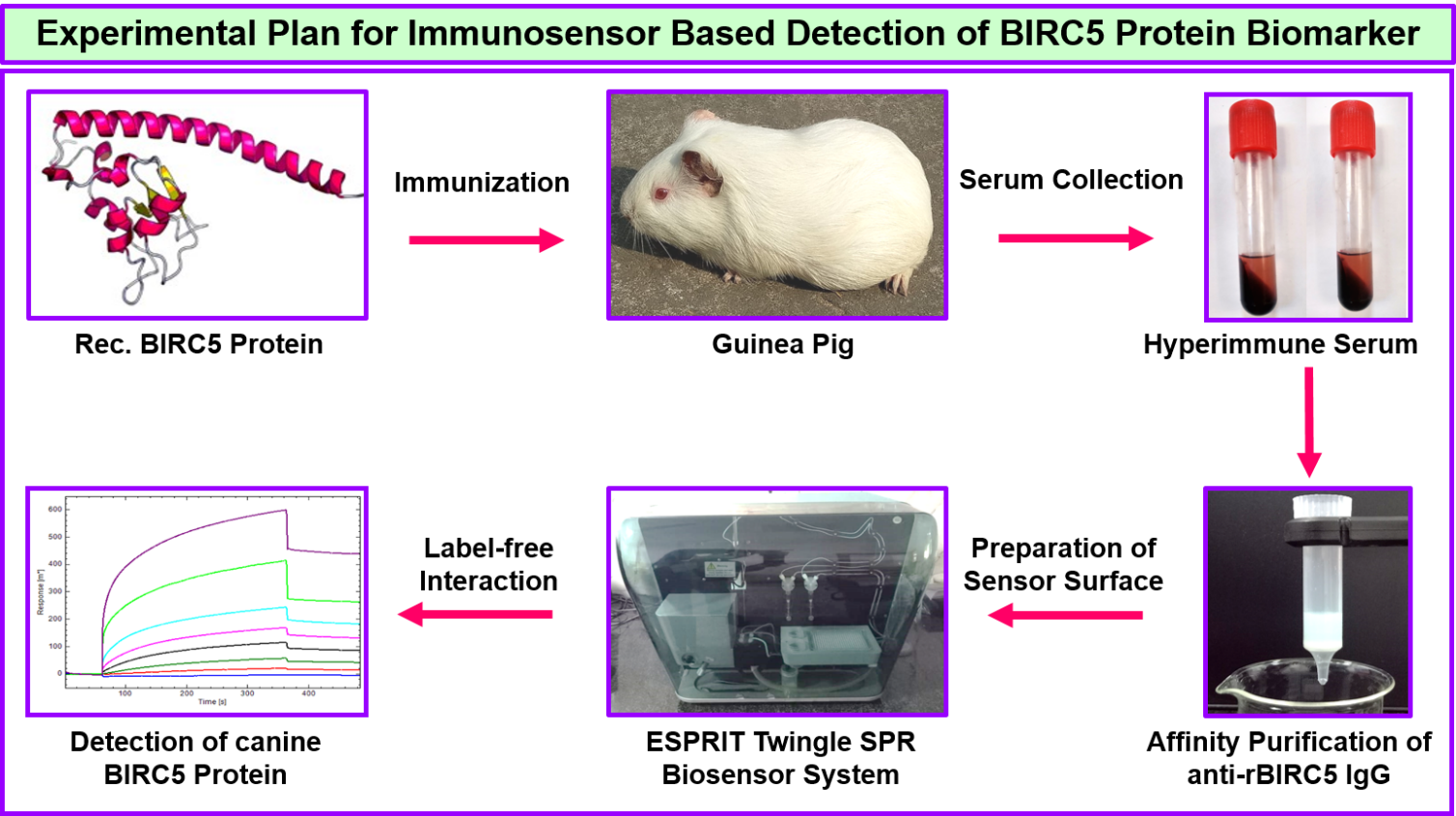


**Figure-S1:** Diagrammatic representation of experimental plan for generating polyclonal anti-BIRC5 antibodies, purification and detection of BIRC5 protein biomarker by label-free SPR immunosensor


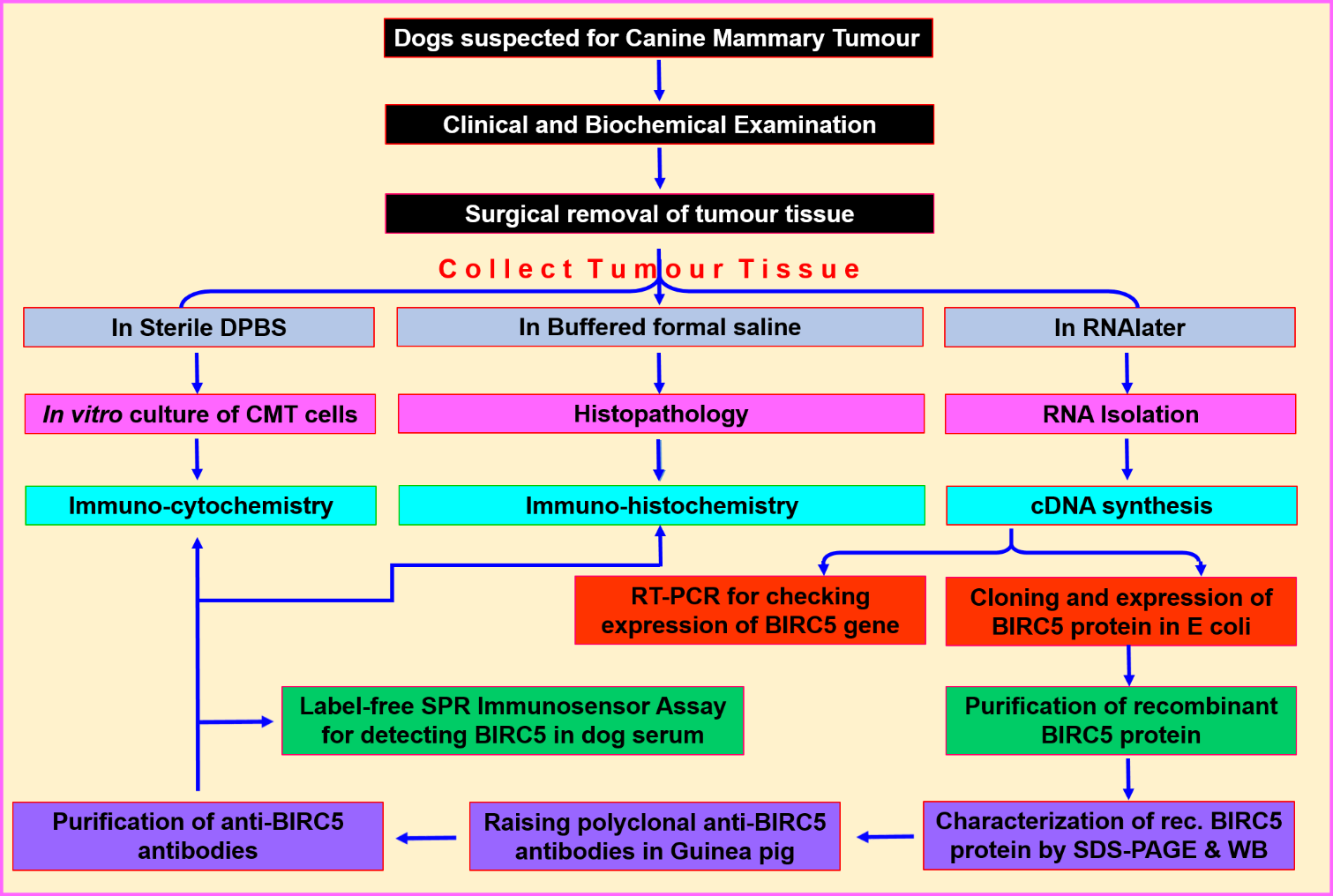


**Figure-S2:** Schematic layout of experimental plan of work for developing SPR immunosensor assay to detect BIRC5 in dog serum


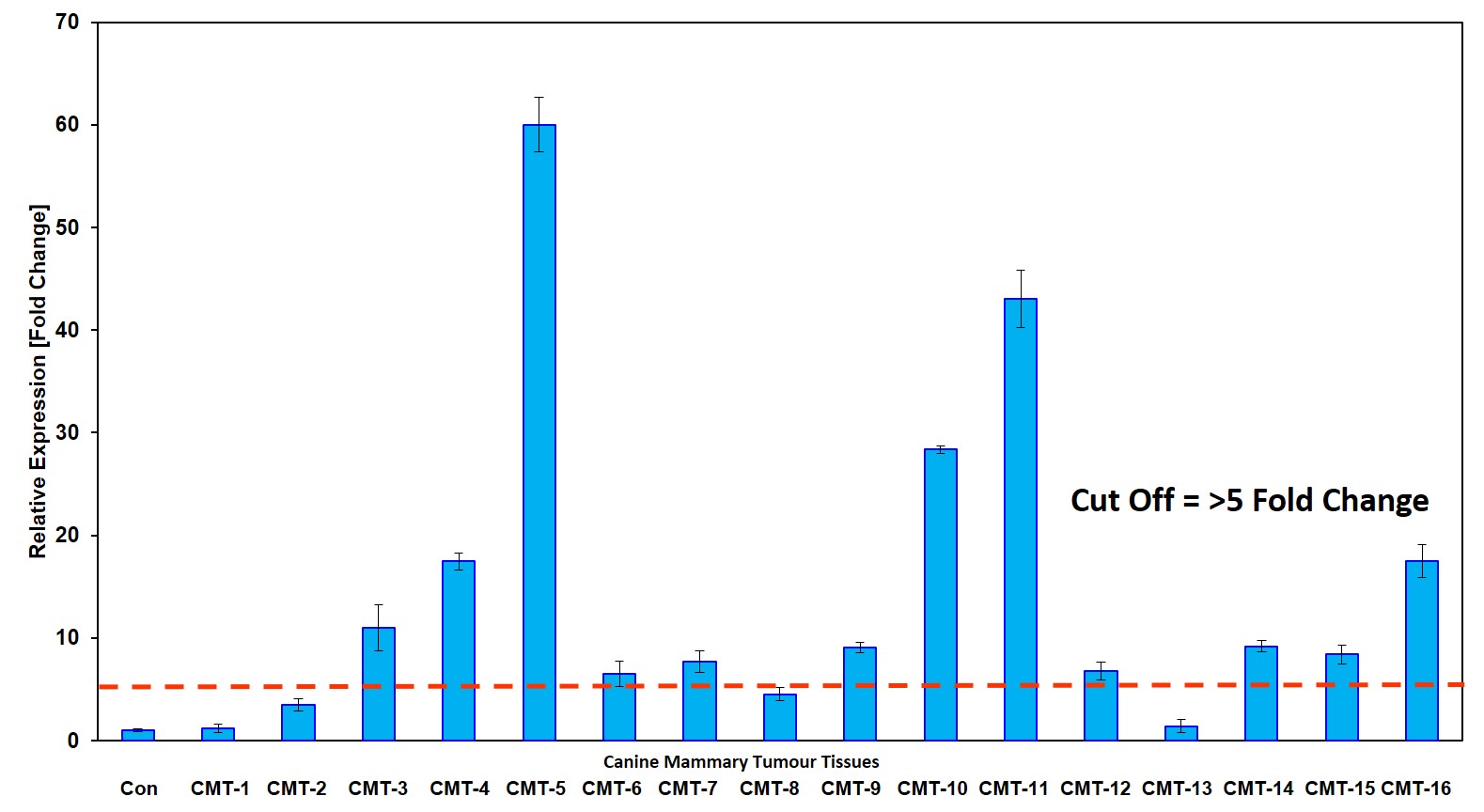


**Figure-S3: Relative expression of BIRC5 in CMT tissues by Real-time PCR:** Over-expression of BIRC5 gene (more than five-fold higher expression) was detected in 12 out of 16 (75%) CMT tissues as compared to normal healthy mammary gland tissues (Con).


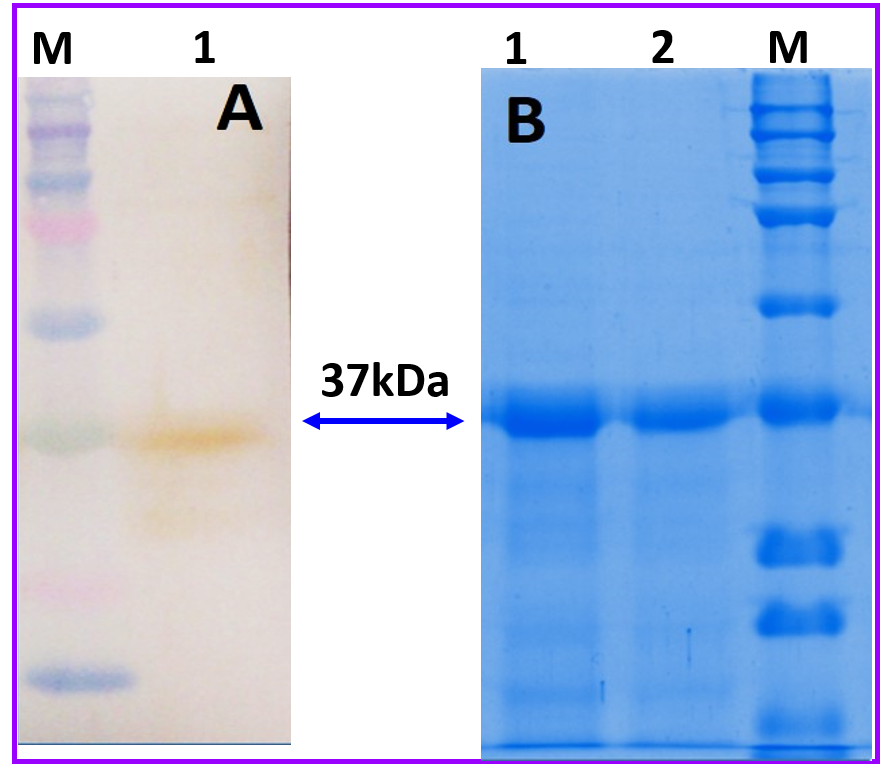


**Figure-S4:** Characterization of recombinant BIRC5 protein (37kDa): [A] Western blot using polyclonal rabbit anti-human BIRC5 (1:200), Lane M: Pre-stained molecular weight marker, Lane 1: Reactivity of purified recombinant protein with specific antibodies [B] SDS-PAGE analysis, Lane M: Unstained protein molecular weight marker, Lanes-1 and 2: Recombinant protein purified by Ni-NTA affinity chromatography using AKTA Pure 25M FPLC system


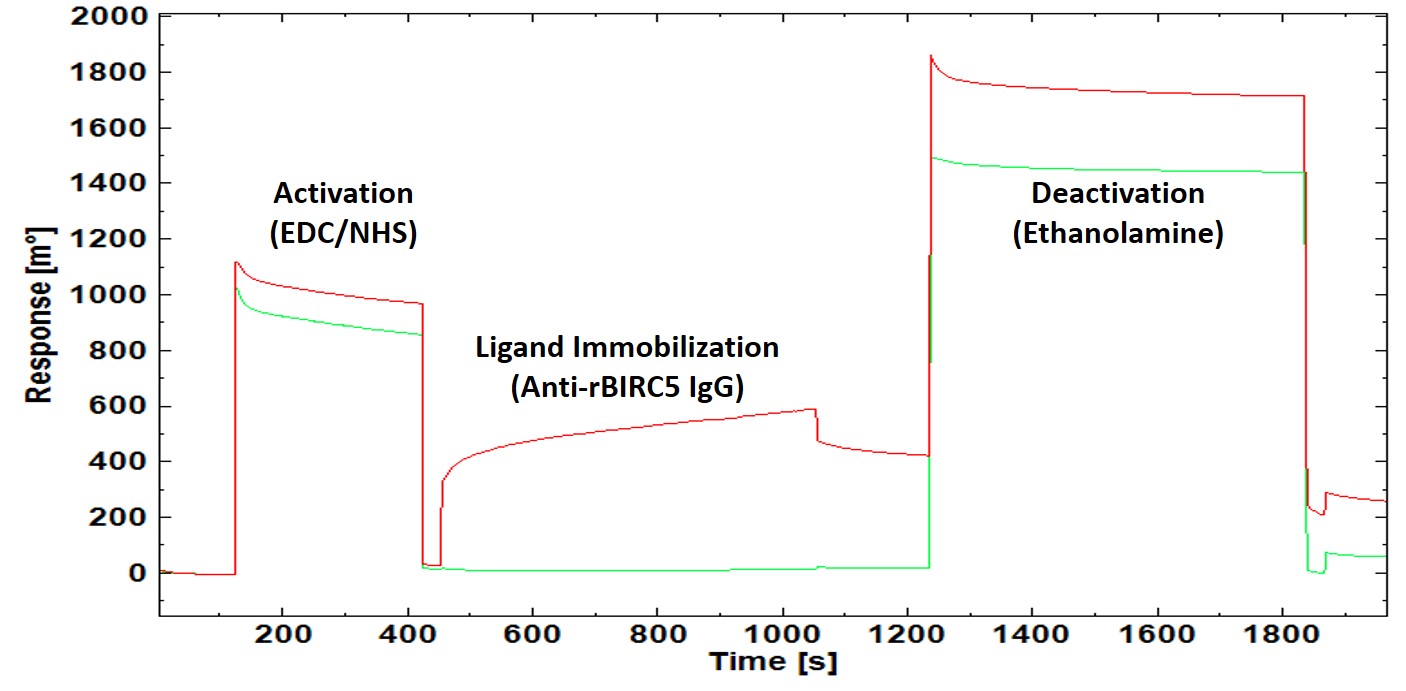


**Figure-S5:** Preparation of SPR sensor surface: Immobilization of purified antibodies (raised in guinea pig against recombinant BIRC5 protein) on sensor surface using EDC/NHS coupling methodology, channel-1 (red): anti-rBIRC5 IgG, channel-2 (green): reference.

**
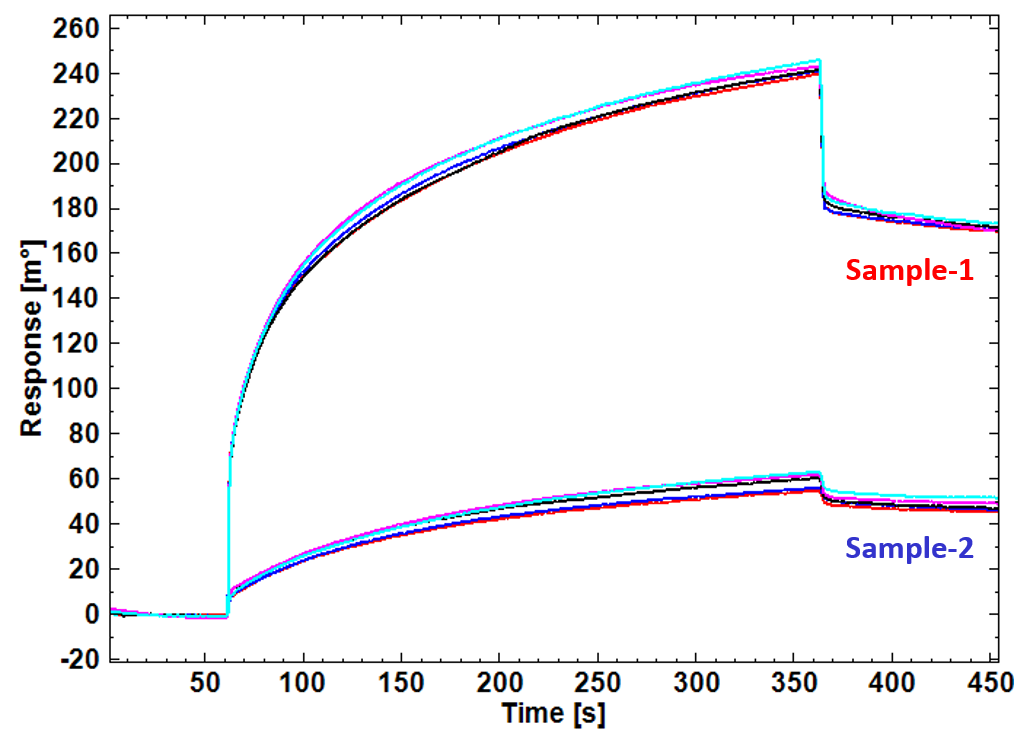
**

**Figure-S6:** Reproducibility of SPR assay results: Overlay of five sensorgrams obtained on interaction of BIRC5 protein present in serum samples with antibodies immobilized on sensor surface. Two different samples were tested on the same sensor chip after treatment with regeneration buffer in each cycle. The assay produced reproducible results and almost similar values were recoded each time for corresponding sample, which also indicate perfect optimization of the regeneration protocol without loss of surface bound antibodies after each cycle of regeneration.


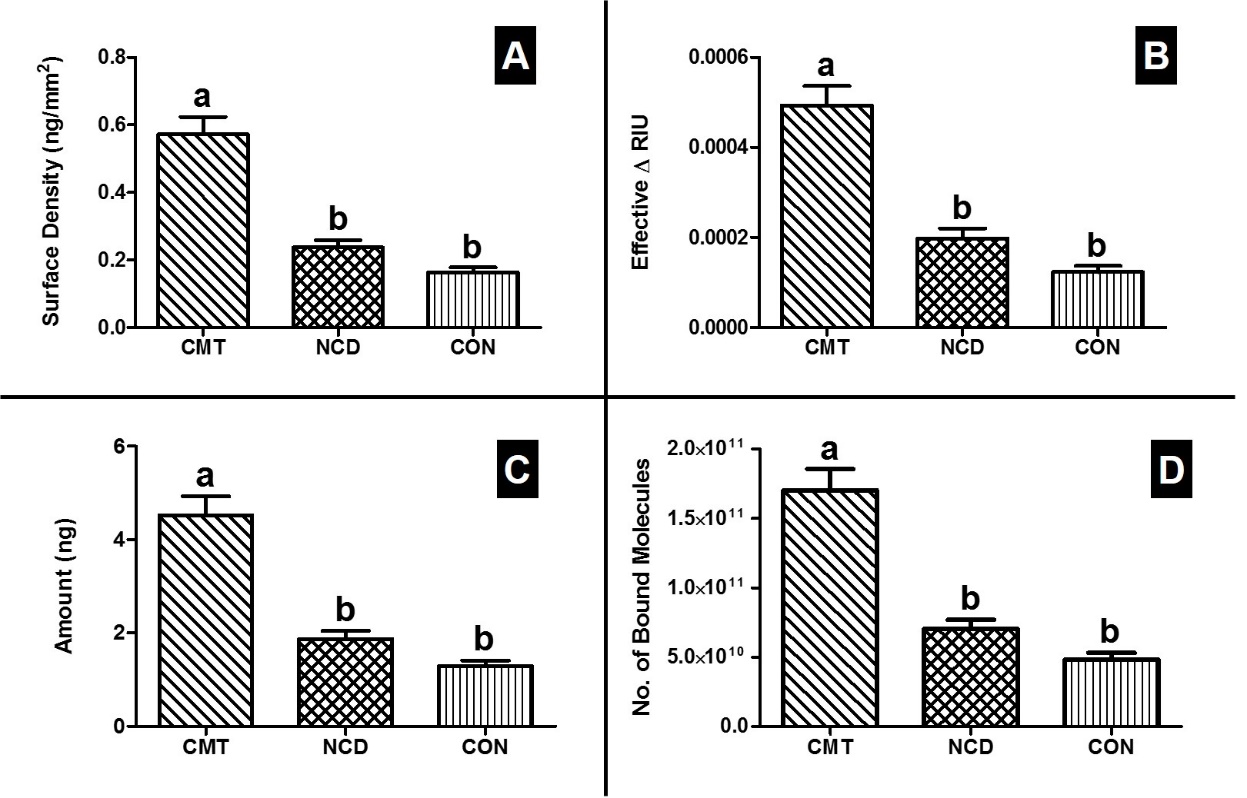


**Figure-S7:** Bar diagrams showing binding parameters (Mean ± SEM) for interaction between BIRC5 protein present in dog sera and specific antibodies immobilized on sensor surface. (A) Surface density (B) Effective shift in Refractive Index Unit (C) Amount of BIRC5 protein and (D) Number of BIRC5 protein molecules bound on the sensor surface

**
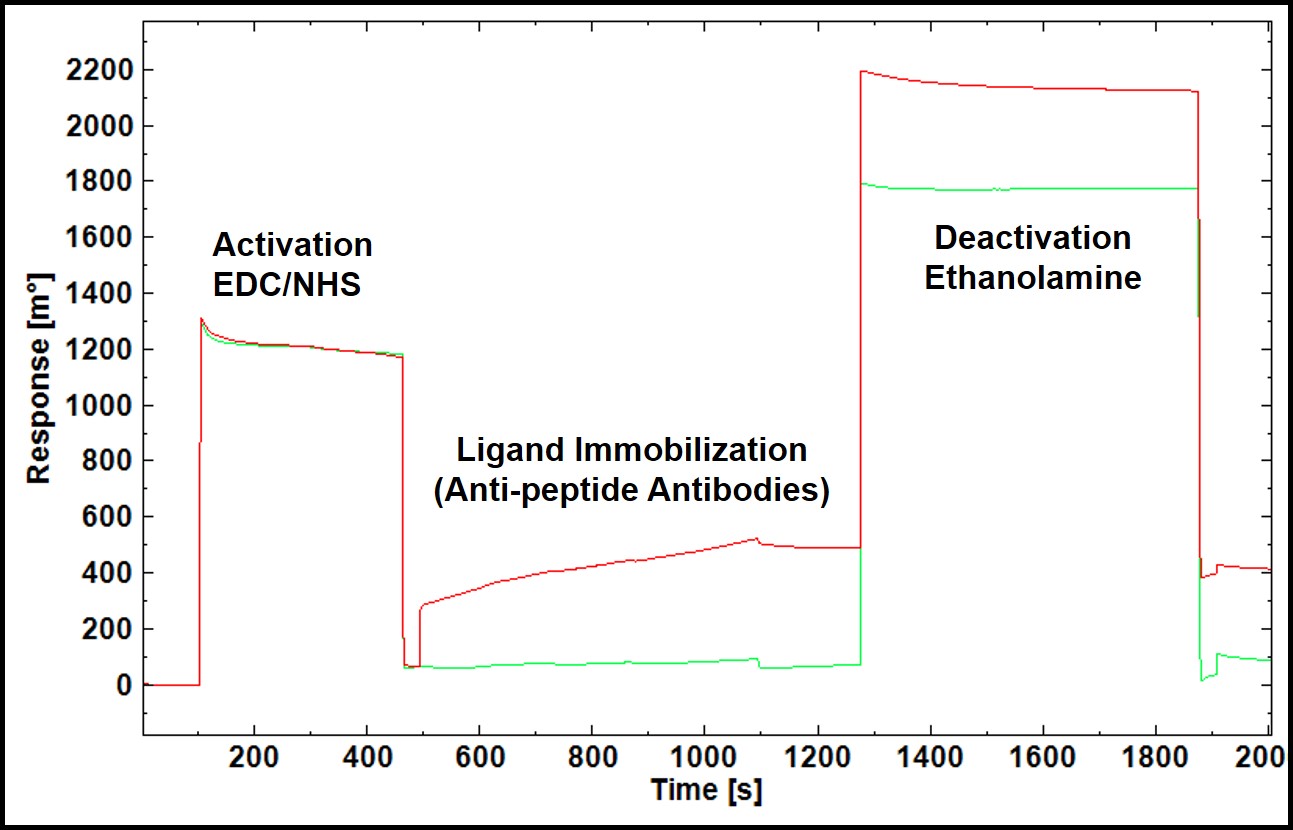
**

**Figure-S8:** Preparation of SPR sensor surface: Immobilization of purified anti-peptide antibodies (raised in guinea pig against synthetic peptide antigen corresponding to viral protein) on sensor surface using EDC/NHS coupling methodology, channel-1 (red): purified anti-peptide antibodies, channel-2 (green): reference.

**
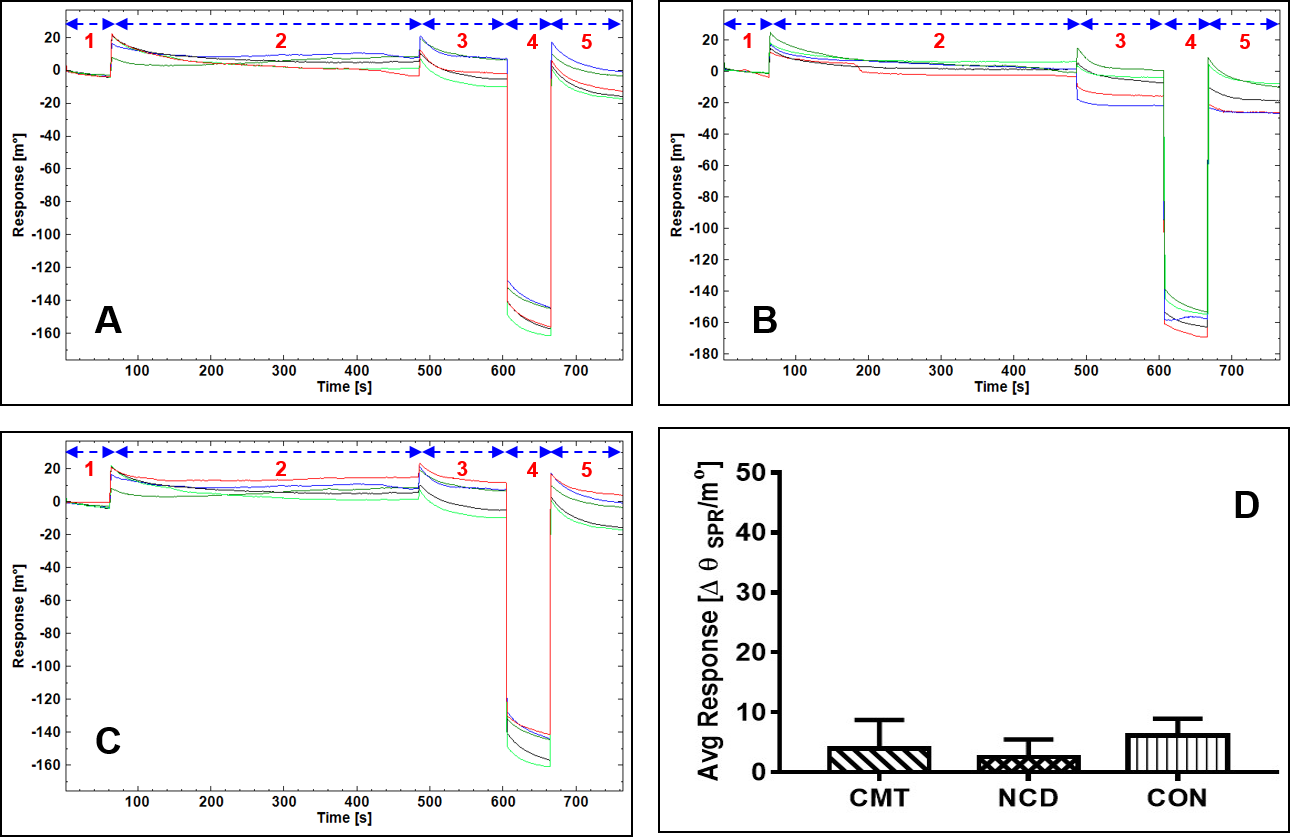
**

**Figure-S9:** Overlay of SPR sensorgrams showing interaction of representative serum samples (n=5, shown in different colors) from (A) CMT, (B) NCD and (C) CON with purified anti-peptide antibodies (raised in guinea pig against synthetic peptide antigen corresponding to viral protein) immobilized on SPR sensor chip. In each sensorgram, sample was injected after obtaining a baseline for 60 sec (shown as 1), association was observed for 420 sec (shown as 2), unbound analyte was removed during dissociation for 120 sec (shown as 3). Thereafter, the sensor chip was regenerated for a second round of interaction by passing regeneration buffer for a period of 60 sec (shown as 4). After regeneration, the chip was again washed with running buffer to obtain the baseline (shown as 5).

(D) Bar diagram showing average SPR response of representative serum samples of each group (n=5 each). These samples showed negligible SPR signals when tested with non-specific antibody immobilized on sensor chip.
